# Antiglycemic Effect of Water Extractable Arabinoxylan from Wheat Aleurone and Bran

**DOI:** 10.1155/2017/5784759

**Published:** 2017-05-24

**Authors:** Lovemore Nkhata Malunga, Marta Izydorczyk, Trust Beta

**Affiliations:** ^1^Department of Food Science, University of Manitoba, Winnipeg, MB, Canada R3T 2N2; ^2^Canadian Grain Commission, Winnipeg, MB, Canada R3C 3G8; ^3^Richardson Centre for Functional Foods & Nutraceuticals, University of Manitoba, Winnipeg, MB, Canada R3T 2N2

## Abstract

The studies on the effects of arabinoxylan (AX) polysaccharides on postprandial glucose response have resulted in contrasting results owing to the diversity in AX structures. Four water extractable AX (WEAX) extracts obtained from wheat aleurone and bran were used to investigate (a) the effect of AX on activities of *α*-amylase and *α*-glucosidase, (b) influence of AX chemical composition on their inhibition potency, and (c) kinetics of enzyme inhibition. *α*-Amylase activity was not significantly affected by the presence WEAX fractions regardless of type or concentration. WEAX inhibited *α*-glucosidase activity only when maltose was used as a substrate but not sucrose. The IC50 values of WEAX (4.88 ± 0.3–10.14 ± 0.5 mg/mL) were highly correlated to ferulic acid content (*R* = −0.89), arabinose to xylose ratio (*R* = −0.67), and relative proportions of xylose being unsubstituted (*R* = 0.69), disubstituted (*R* = −0.63), and monosubstituted (*R* = −0.76). The Lineweaver–Burk plot suggested an uncompetitive enzyme inhibition mode. Thus, our results suggest that antiglycemic properties of WEAX may be derived from direct inhibition of *α*-glucosidase activity.

## 1. Introduction

The prevalence of type 2 diabetes is increasing globally. Diabetes is a chronic disease epitomised by high circulating plasma glucose. Thus management of postprandial glucose is critical in prevention and treatment of type 2 diabetes patients. Human intervention studies have shown that consumption of an arabinoxylan- (AX-) rich diet attenuates postprandial blood glucose levels in healthy, impaired glucose tolerance, and diabetic subjects [1–5]. In contrast, Mohlig and coworkers [6] found no effect on glucose response when healthy human subjects were fed bread rolls supplemented with AX. Animal studies have also reported mixed results on effect of AX supplementation [7, 8]. The underlying mechanisms remain unclear but it is purported that soluble fibers increase lumen viscosity thereby delaying nutrient absorption [9]. The apparent viscosity of AX solutions is affected by the asymmetrical conformation and molecular weight of AXs as well as the polymer concentration [10]. Of these three factors, concentration of AX seems to influence the viscosity the most [11]. Thus the effect of AXs on blood glucose is dose-dependent. Apparent viscosity of the AX solutions is also dependent on shear stress such that higher shear results in shear thinning, the behaviour characteristic for non-Newtonian fluids [10]. Recent studies suggest that viscosity effect of AX may be offset by strong intestinal peristalsis [12].

The molecular structure of AX is complex and heterogeneous. AXs consist of the xylan backbone made of (1 → 4) linked *β*-D-xylopyranosyl (Xyl*p*) residues with *α*-L-arabinofuranosyl (Ara*f*) residues linked to the xylan backbone at C(O)-2 and C(O)-3 and/or at both C(O)-2 and C(O)-3 positions [10]. Xylose residues may also be substituted with glucuronic acid and/or methylglucuronic acid linkages [13]. Ferulic or coumaric acid residues are ester linked to arabinose residues at C(O)-5 position [14, 15]. The ratio of arabinose to xylose, pattern of arabinose substitution, degree of feruloylation, and molecular weight vary greatly among and within cereal grains [10]. Arabinoxylans (AX) constitute the highest proportion of dietary fiber in cereals grains (60−70%) [16] and their content varies with source or grain fraction. AX accounts for 1.3–2.7% w/w of wheat [17]. Wheat aleurone and pericarp contain 20 and 45% AX, respectively [16]. Much of the AXs in wheat are water insoluble (70−86%) [17].

Dietary digestible carbohydrates are hydrolysed to the monomeric sugars, glucose, or fructose prior to their absorption in gastrointestinal tract [18]. Starch is digested primarily to maltose and other short chain carbohydrates by salivary and pancreatic amylase. The resultant (maltose, maltotriose, and *α*-limit dextrins) and sucrose are digested to glucose or fructose by the small intestinal brush border *α*-glucosidases (maltase-glucoamylase and sucrase-isomaltase) [19]. Sugar absorption in the small intestine mainly involves GLUT2, GLUT5, and SGLT1 transporters [20, 21]. Thus decrease in postprandial hyperglycaemia can be attained by limiting intestinal carbohydrate digestion or uptake. Despite the enormous differences in structure of AX, most studies report very little or no detail of composition or structure of the AX used making it difficult to compare the results on effect of AX on postprandial blood glucose [22]. Very limited data also exist on effect of purified water extractable AXs on carbohydrate digestive enzymes. Thus in this study we aimed at investigating (a) the effect of AX on activities of *α*-amylase and *α*-glucosidase, (b) the influence of AX chemical composition on their inhibition potency, and (c) the kinetics of enzyme inhibition.

## 2. Materials and Methods

### 2.1. Chemicals and Reagents

A commercial wheat aleurone (Grainwise wheat aleurone) was a gift from Cargill Limited and Horizon Milling (Wichita, Kansas, USA). It consists of 4.5, 15.2, 7.4, and 2.5% lipid, protein, ash, and starch, respectively. Hard red winter wheat bran was purchased locally from Bulk Barn (Winnipeg, Manitoba, Canada). Its moisture, ash, and protein content were analysed to be 5.8, 5.3, and 11.1%, respectively. Wheat unmodified starch, maltose, sucrose, acarbose, porcine pancreas *α*-amylase (EC 3.2.1.1, Type VI-B), amyloglucosidase (EC 3.2.1.3) from aspergillus, and intestinal acetone powders from rat were bought from Sigma-Aldrich (Milwaukee, WI, USA). Ammonium sulphate, all acids, and organic solvents were bought from Fischer Scientific (Whitby, Ontario, Canada). Maltose, sucrose, and glucose assay kit (K-MASUG 08/13) was purchased from Megazyme International Ireland (Bray, Wicklow, Ireland). All chemicals used were of analytical or HPLC grade.

### 2.2. Water Extractable Arabinoxylan Preparation

The endogenous enzymes were inactivated by boiling wheat bran and wheat aleurone samples (~200 g) in 2 L aqueous ethanol (80%, v/v) at 85°C under reflux for 2 hours. The supernatant was discarded and the residue was air-dried in a fume-hood overnight at room temperature. Water extractable fractions were isolated from the air-dried bran or aleurone (150 g) at 45°C according to the method described by Izydorczyk and Biliaderis [23]. The water extract was destarched using *α*-amylase (1821 U/L) and deproteinized by celite and fuller's earth. The purified material was fractionated by graded ammonium sulphate (AS) precipitation and fractions were obtained at 50 and 75% AS saturation. The material collected was freeze dried after being dialyzed (12 kDa cut-off membrane) for 48 hours. Water extractable fractions collected from wheat aleurone were labeled (WA) followed by the concentration of AS at which they were obtained (WA-f50 and WA-f75). Similarly, the collected materials from wheat bran (WB) were designated as WB-f50 and WB-f75. The chemical and structural descriptions of WEAX fractions are presented in Table 1.

### 2.3. Inhibition Assay for *α*-Amylase Activity

Wheat starch (300 mg) was suspended in 15 mL sodium phosphate buffer (pH 6.9, 0.1 M) containing 1 mM calcium chloride and cooked at 95°C for 15 minutes [12]. WEAX fractions (40 mg) were dissolved in 2 mL sodium phosphate buffer. Samples were diluted such that the final concentration in the reaction mixture was 0.0, 0.2, 0.3, and 0.5% (w/v). Equal volumes (200 *μ*L) of starch and WEAX (or control) were mixed and vortexed. Starch hydrolysis was initiated by adding 70 *μ*L of porcine pancreatic *α*-amylase (130 U/mL) and 40 *μ*L of fungal amyloglucosidase (240 U/mL). The reaction was stopped after 30 minutes by heating at 95°C for 5 minutes. The mixture was immediately cooled on ice and centrifuged (Thermo Scientific, Sorvall Legend Micro21, Germany). The supernatants were collected and analysed for glucose using Megazyme glucose test kit. Human intervention studies have reported the effectiveness of a 0.25 to 0.70% AX concentration [1–3] and hence our choice of the concentration range.

### 2.4. Inhibition Assay for Rat Intestinal *α*-Glucosidase Activity

The *α*-glucosidase inhibitory method by Oki et al. [24] was used with modifications. Briefly, rat intestinal acetone powder (500 mg) was mixed with 10 mL sodium phosphate buffer (pH 6.9, 0.1 M) and sonicated in ice bath for 30 seconds (12 times) with 15 seconds break to prevent heat buildup. The mixture was later centrifuged at 10000*g* at 4°C for 10 minutes. The supernatant was collected and labeled rat intestinal *α*-glucosidase. WEAX samples (40 mg) were dissolved in 2 mL sodium phosphate buffer (pH 6.9, 0.1 M). Later, 50 *μ*L rat intestinal *α*-glucosidase was mixed with 100 *μ*L sample or buffer (control) and incubated at 37°C for 5 minutes. Fifty *μ*L of 20 mM sucrose or 4 mM maltose was added and further incubated for 60 minutes (sucrose) or 30 minutes (maltose). Final WEAX fraction concentration was 0.5, 0.4, 0.3, 0.2, 0.1, and 0.0% (w/v). The enzyme activity was halted by heating at 95°C for 10 minutes. After centrifugation at 10000*g* for 10 minutes, the supernatants were collected for glucose analysis using the Megazyme GOPOD glucose test kit. Alpha glucosidase (sucrase or maltase) inhibition % was calculated as (1–[(*A*_sample_–*A*_blank_)/(*A*_control_ − *A*_blank_)])*∗*100. IC50 value was determined from the plot of %  *α*-glucosidase inhibition against sample concentration. Inhibition of rat intestinal *α*-glucosidase with acarbose (a known *α*-glucosidase inhibitor) was also done for comparison purpose. Acarbose concentrations of 1.625, 3.25, 4.9, 6.5, 9.8, and 13 *μ*g/mL were used instead of sample.

### 2.5. Statistical Analysis

All analyses were performed in sextuplicate (unless indicated otherwise) and all statistics were calculated using one way analysis of variance (ANOVA) on a JMP 12 statistical software (SAS Institute Inc., Cary, NC). Sample means were compared using Tukey HSD method and significant differences determined at *p* ≤ 0.05. Correlations among parameters were computed using Pearson's correlation test.

## 3. Results and Discussion

**Figure 1:**
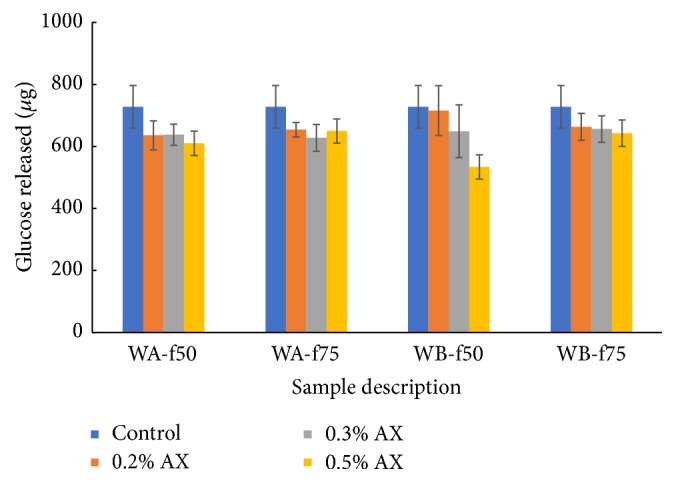
Effect of water extractable arabinoxylan on starch hydrolysis.

The effects of WEAX on starch hydrolysis are presented in Figure 1. We compared the amount of glucose released over 30 minutes of incubation with *α*-amylase in the presence or absence of WEAX fractions. Addition of WEAX fractions numerically decreased the amount of glucose produced compared to control treatment. However, statistical comparisons of treatment groups and control showed that the mean difference was not significant (*p* < 0.05) for WA-f_50_, WA-f_75_, and WB-f_75_ regardless of WEAX concentration. The presence of 0.5% WB-f_50_ resulted in significant decrease in amylolysis compared to control (*p* < 0.05). However, our observations for alpha amylase activity contrasted other reports in literature possibly due to concentration and type of AX. Amylolysis of starch was performed in the presence of 1 and 2% AX and the AX used was devoid of ferulic acid [12]. We used concentrations of AX (~5–10 g) equivalent to that reported to attenuate postprandial blood glucose in human studies [1–3].

Table 2 shows the effect of WEAX on *α*-glucosidase activity in the presence of sucrose or maltose as substrate. The data is presented as IC50 which is the concentration of the inhibitor resulting in 50% inhibition of *α*-glucosidase activity. The IC50 values ranged from 4.88–10.14 mg/mL against *α*-glucosidase activity with maltose as substrate. However, no inhibition was observed when sucrose was used as a substrate. The inhibitory potency of WEAX against intestinal maltase was 1000–2000 times less compared to acarbose (a positive control). It is widely hypothesised that viscosity may be the cause of the effect of AX on postprandial glucose [22]. Thus Vogel et al. fed rats a modified wheat bran AX to study the effect of viscosity [8 AXs were modified through oxidative gelation to increase their viscosity. Rats fed on modified AX had significantly lower postprandial blood glucose but not those fed on native AX demonstrating the role of viscosity. However, in this case, we used similar concentration of WEAX in both *α*-amylase and *α*-glucosidase activity studies and yet only the later was inhibited. This may imply that the effect of WEAX fractions on *α*-glucosidase activity may not have been a consequence of viscosity but rather substrate-enzyme-inhibitor interaction. The same is supported by the observation that sucrose activity of *α*-glucosidase was not affected by the presence of WEAX in our study. Thus it is possible that the antiglycemic effect of AX may be due to inhibition of *α*-glucosidase activity. Even though its potency on *α*-glucosidase activity is 1000–2000 times less compared to acarbose, IC50 concentration is achievable upon consumption recommended dietary requirement of fiber (21–38 g/day).

The inhibition potency varied significantly (*p* < 0.05) among the WEAX samples with WA-f_50_ being the most potent. WEAX isolated at 50% ammonium sulphate saturation exhibited higher inhibition capacity compared to their corresponding fractions obtained at 75%. A Pearson correlation analysis (Table 3) suggested that ferulic acid content, arabinose to xylose ratio, and pattern of xylose substitution may have influenced the inhibition activity of WEAX. Ferulic acid content of AX was a major determinant (*R* = −0.89) of their inhibition potency. Antidiabetic properties of plant extracts have been associated with their total phenolic compound content [25, 26]. Also, inhibition of intestinal alpha glucosidase by AX mono-/oligosaccharide was also related to their ferulic acid moiety [27]. Hence, WA-f_50_ and WB-f_75_ had significantly different inhibition potency towards *α*-glucosidase activity despite having similar relative proportions unsubstituted (un-Xyl*p*), monosubstituted, (2-Xyl*p* or 3-Xyl*p*), and disubstituted xylose residues (2,3-Xyl*p*) and degree of substitution but different ferulic acid content.

Arabinose to xylose ratio is a measure of degree of substitution (DS). A strong negative linear association (*R* = −0.67) was observed between DS and IC50. This could have been a consequence of increased solubility of AX due to high DS. Thus highly substituted WEAX seems to have lower IC50 value (high inhibition potency). The same observation was supported by negative association between inhibition potency and relative proportion of unsubstituted xylose residues. The apparent di-Xyl_p_ to mono-Xyl_p_ residues ratio did not seem to have influence but the extent of xylose substitution showed an effect. Thus it is likely that the effect on *α*-glucosidase activity may emanate from arabinose residues of WEAX. There have been reports on arabinose inhibiting *α*-glucosidase activity [28]. Attempts to remove arabinose residues from WEAX using arabinofuranosidase were not successful in order to prove the hypothesis. However, we noted that mono-Xyl_p_ at C (O)-2 was a major determinant compared to mono-Xyl_p_ at C (O)-3 suggesting that inhibition potency was beyond the mere presence of arabinose residue. There was a strong correlation between mono-Xyl_p_ at C (O)-2 and ferulic acid content (*R* = 0.99). Thus it is possible that the observed influence of DS could have been derived from that of ferulic acid.

**Figure 2:**
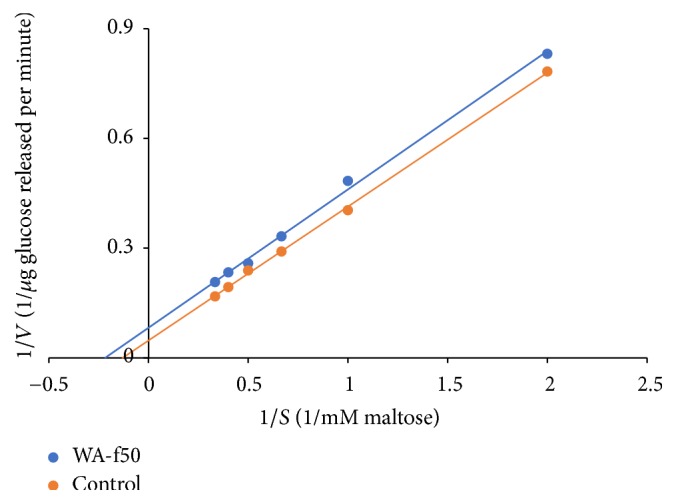
Lineweaver–Burk plot of rat intestinal *α*-glucosidase inhibition by water extractable arabinoxylan (WA_-f50_).

The Lineweaver–Burk plot (Figure 2) was used to calculate the apparent maximum velocity (*V*_max_) and Michaelis–Menten constant (*K*_m_) for *α*-glucosidase activity on maltose in the presence and absence of WEAX. The effect of WEAX fraction on *V*_max_ and *K*_m_ was analysed to determine the type of inhibition. *V*_max_ and *K*_m_ of *α*-glucosidase for maltose in absence of WEAX fractions were 17.5 *μ*g glucose per minute and 5.99 mM, respectively.  Table 4 shows that addition of WEAX fractions decreased both *V*_max_ and *K*_m_ values suggesting that WEAX inhibited *α*-glucosidase activity through an uncompetitive mode. A typical characteristic of uncompetitive inhibition is that both *V*_max_ and *K*_m_ decrease in the presence of inhibitor. Thus it is plausible that WEAX fractions bind to enzyme substrate complex thereby decreasing both *V*_max_ and *K*_m_. Arabinose was also found to inhibit *α*-glucosidase activity through an uncompetitive mode [28].

Our results may provide an explanation on the inconsistency observed in literature about the effect of AX on postprandial glucose level. Feeding of Zuker diabetic rats with AX (Ara/Xyl = 0.9) supplemented bread resulted in a significant decrease in postprandial blood glucose level [7]. In contrast, intake of native AX (Ara/Xyl = 0.5) had no effect on blood glucose response [8]. Also, supplementation of diets with 6 and 12 g AX (Ara/Xyl = 0.66 or 0.8) decreased blood glucose in both healthy and diabetic subjects [1, 2, 5]. However, AX (Ara/Xyl = 0.8) did not attenuate postprandial glucose response in healthy human adults [6]. Pigs fed on white bread supplemented with AXs had a reduced net glucose flux compared to pigs fed on white bread [29]. The absence of detailed chemical composition and structures makes it difficult to compare the results on effectiveness of AX [22]. Thus even though concentrations of AXs used may be the same, its effectiveness would depend on the nature of AXs used. We have demonstrated that AX obtained at 50% ammonium sulphate saturation exhibited higher inhibition potency compared to AX obtained at 75%.

## 4. Conclusion

The results of this study indicated that antiglycemic effect of arabinoxylans may be derived from inhibiting intestinal *α*-glucosidase activity but not amylase activity. The potency of water extractable AX on *α*-glucosidase activity was influenced by ferulic acid contents arabinose to xylose ratio and pattern of xylose substitution. The findings also suggest that inhibition of *α*-glucosidase activity occurs through uncompetitive mechanism. Thus, consumption of diet rich in water extractable AX may attenuate postprandial blood glucose level.

## Figures and Tables

**Table 1:** Chemical composition and characteristics of water extractable arabinoxylan from wheat aleurone (WA) and wheat bran (WB).

	Wheat aleurone		Wheat bran	
	WA-f_50_	WA-f_75_	WB-f_50_	WB-f_75_
Total carbohydrate content (% w/w)	76.0 ± 0.6	85.7 ± 1.0	54.6 ± 0.5	81.6 ± 1.3
Protein content (% w/w)	8.7 ± 0.2	8.6 ± 0.2	16.9 ± 0.3	11.3 ± 0.3
Beta glucan content (% w/w)	0.4	0.7	12.5	15.7
Arabinoxylan content (% w/w)	74.0	83.9	41.5	66.6
Arabinose to xylose ratio	0.58	0.44	0.85	0.56
Total ferulic acid content	26.01 ± 0.40	6.53 ± 0.20	16.78 ± 0.35	4.34 ± 0.11
Uronic acid content (%)	0.04 ± 0.0	0.05 ± 0.0	0.08 ± 0.0	0.10 ± 0.0
Average molecular weight (kDa)	551.0	677.0	643.0	468.0
*Patten of substitution*				
Unsub- Xylp (%)	61	70	45.1	63.8
Mono-Xylp at C (O)-2 (%)	3.2	0.2	1.4	0.1
Mono-Xylp at C (O)-3 (%)	16.8	15.9	23.4	16.4
Total mono-Xylp (%)	20.1	16.1	24.8	16.5
Di-Xylp (%)	19.0	14.0	30.1	19.8

Values presented as mean ± standard deviation (*n* = 3). Un-Xyl*p*: unsubstituted xylose residues, mono-Xyl*p*: monosubstituted xylose residue,di-Xyl*p*: O-2 and O-3 disubstituted xylose residues.WA-f_50_ and WA-f_75_: water-extractable fractions from WA obtained at 50 and 75% ammonium sulphate saturation, respectively. WB-f_50_ and WB-f_75_: water-extractable from WB obtained at 50 and 75% ammonium sulphate saturation, respectively.

**Table 2:** Effect of feruloylated arabinoxylan on intestinal *α*-glucosidase when sucrose or maltose was used as substrate.

	IC50 (mg/mL)	
	Maltose	Sucrose
WA-f_50_	4.88 ± 0.30^d^ (0.49)	*∗*
WA-f_75_	10.14 ± 0.56^a^ (1.01)	*∗*
WB-f_50_	5.73 ± 0.19^c^ (0.57)	*∗*
WB-f_75_	8.15 ± 0.46^b^ (0.81)	*∗*
Acarbose	0.005 ± 0.00^e^ (~0.0005)	0.003 ± 0.00

Values presented as mean ± standard deviation (*n* = 6). Data in the same column with the same superscript are not significantly different at *p* ≤ 0.05. Data in parenthesis are IC50 values in % w/v. IC50 value is the sample concentration resulting in 50% inhibition of *α*-glucosidase activity. WA-f_50_ and WA-f_75_: water-extractable fractions from WA obtained at 50 and 75% ammonium sulphate saturation, respectively. WB-f_50_ and WB-f_75_: water-extractable from WB obtained at 50 and 75% ammonium sulphate saturation, respectively.

**Table 3:** Correlation coefficient of arabinoxylans' inhibition of *α*-glucosidase activity and its structural properties.

	*α*-Glucosidase activity (IC50)
Arabinose to xylose ratio	−0.67
Ferulic acid content	−0.89
Unsub-Xyl*p*	0.69
Mono-Xyl*p* at C (O)-32	−0.86
Mono-Xyl*p* at C (O)-3	−0.51
Total Mono-Xyl*p*	−0.76
Di-Xyl*p*	−0.63
Molecular weight	0.23
Uronic acid	0.36

Data represent Pearson correlation coefficient values at *p* ≤ 0.05. un-Xyl*p*: unsubstituted xylose residues, mono-Xyl*p*: monosubstituted xylose residue, di-Xyl*p*: C (O)-2 and C (O)-3 disubstituted xylose residues.

**Table 4:** Inhibition kinetics of water extractable arabinoxylans derived from Lineweaver–Burk plots.

	*V*_max_ (*µ*g glucose/minute)	*K*_m_ (mM maltose)
Control	17.5 ± 0.48^a^	5.99 ± 0.16^a^
WA-f_50_	12.07 ± 0.22^c^	4.54 ± 0.08^c^
WA-f_75_	16.73 ± 0.42^a^	5.07 ± 0.13^b^
WB-f_50_	Nd	nd
WB-f_75_	14.73 ± 0.33^b^	4.91 ± 0.11^b^

Values presented as mean ± standard deviation (*n* = 3). Data in the same column with the same superscript are not significantly different at *p* ≤ 0.05. *V*_max_ = maximum velocity; *K*_m_ is the Michalelis–Menten constant (substrate concentration required for an enzyme to reach half *V*_max_). nd means not determined. WA-f_50_ and WA-f_75_: water-extractable fractions from WA obtained at 50 and 75% ammonium sulphate saturation, respectively. WB-f_50_ and WB-f_75_: water-extractable from WB obtained at 50 and 75% ammonium sulphate saturation, respectively.
